# Psychologically based interventions for adults with chronic neuropathic pain: a scoping review

**DOI:** 10.1093/pm/pnae006

**Published:** 2024-02-04

**Authors:** Mayumi Oguchi, Michael K Nicholas, Ali Asghari, Duncan Sanders, Paul J Wrigley

**Affiliations:** Sydney Medical School—Northern, Faculty of Medicine and Health, University of Sydney, St Leonards, NSW 2065, Australia; Pain Management Research Institute, Kolling Institute, Northern Sydney Local Health District and the Faculty of Medicine and Health, The University of Sydney, St Leonards, NSW 2065, Australia; Pain Management and Research Centre, Royal North Shore Hospital, St Leonards, NSW 2065, Australia; Sydney Medical School—Northern, Faculty of Medicine and Health, University of Sydney, St Leonards, NSW 2065, Australia; Pain Management Research Institute, Kolling Institute, Northern Sydney Local Health District and the Faculty of Medicine and Health, The University of Sydney, St Leonards, NSW 2065, Australia; Pain Management and Research Centre, Royal North Shore Hospital, St Leonards, NSW 2065, Australia; Sydney Medical School—Northern, Faculty of Medicine and Health, University of Sydney, St Leonards, NSW 2065, Australia; Pain Management Research Institute, Kolling Institute, Northern Sydney Local Health District and the Faculty of Medicine and Health, The University of Sydney, St Leonards, NSW 2065, Australia; Pain Management and Research Centre, Royal North Shore Hospital, St Leonards, NSW 2065, Australia; Sydney Medical School—Northern, Faculty of Medicine and Health, University of Sydney, St Leonards, NSW 2065, Australia; Pain Management Research Institute, Kolling Institute, Northern Sydney Local Health District and the Faculty of Medicine and Health, The University of Sydney, St Leonards, NSW 2065, Australia; Sydney Medical School—Northern, Faculty of Medicine and Health, University of Sydney, St Leonards, NSW 2065, Australia; Pain Management Research Institute, Kolling Institute, Northern Sydney Local Health District and the Faculty of Medicine and Health, The University of Sydney, St Leonards, NSW 2065, Australia; Pain Management and Research Centre, Royal North Shore Hospital, St Leonards, NSW 2065, Australia

**Keywords:** scoping review, psychological intervention, neuropathic pain, chronic pain, biopsychosocial perspective

## Abstract

**Objective:**

As psychologically based interventions have been shown to have clinical utility for adults with chronic pain generally, a similar benefit might be expected in the management of chronic neuropathic pain (NeuP). However, to date, this has not been established, with existing systematic reviews on this topic being hampered by the scarcity of randomized controlled trials (RCTs). This review aimed to identify the type of psychologically based interventions studied for adults with chronic NeuP. It also aimed to assess whether there are enough RCTs to justify undertaking an updated systematic review.

**Methods:**

Seven databases and 2 clinical trial registries were searched for NeuP and psychologically based interventions from database inception to December 2021, and the search was updated in February 2023. The search was broadened by reviewing the reference list of included studies and contacting field experts. Predetermined study characteristics were extracted.

**Results:**

Of 4682 records screened, 33 articles (less than 1%) met the eligibility criteria. Four broad intervention approaches were observed, including cognitive-behavioral approaches (*n* = 16), mindfulness/meditation (*n* = 10), trauma-focused therapy (*n* = 4), and hypnosis (*n* = 3). Thirteen RCTs were identified, and of these, 9 retained 20 participants in each arm after treatment.

**Conclusions:**

Cognitive-behavioral therapy was the most common therapeutic approach identified, whereas mindfulness/meditation was the most frequently used technique. Almost half to two-thirds of the studies reported significant improvements in pain, disability, or distress, which suggests that psychologically based interventions are potentially beneficial for adults with chronic NeuP. An updated systematic review seems warranted.

**Study registration:**

Open Science Framework (https://osf.io) (December 6, 2021; DOI: 10.17605/OSF.IO/WNSTM).

## Introduction

Neuropathic pain (NeuP) is defined as pain resulting from “a lesion or disease of the somatosensory nervous system.”^1^^,^^2^ The 11th version of the *International Classification of Diseases* (ICD-11) holds that all chronic pain conditions should be viewed within a biopsychosocial framework.^3^ Accordingly, Treede et al.^3^ argued that chronic pain severity should be coded in terms of pain intensity, distress, and disability. NeuP is difficult to treat with pharmacotherapies alone, and multidisciplinary approaches have been recommended.^4^ Commonly, multidisciplinary treatments include psychologically based methods, and these have been well established in the general chronic pain literature but not in the NeuP literature.^5^^,^^6^ It has been reported that individuals with NeuP are less likely to benefit from multidisciplinary treatments than are people with other types of chronic pain.^7^ It has also been suggested that treatments for individuals with NeuP might need to be tailored differently from treatments for people with other types of chronic pain.^8^^,^^9^ To further complicate matters, there is a lack of good-quality randomized controlled trials (RCTs) that examine the efficacy of psychologically based interventions for the population with chronic NeuP.^10^^,^^11^ In summary, relative to studies of people with heterogeneous chronic pain conditions, little is known about the extent, nature, and efficacy of psychologically based interventions in adults with chronic NeuP. The 2020 Cochrane systematic review of psychologically based treatments for chronic pain included 1 RCT of NeuP,^5^^,^^12^ and the most recent systematic review of RCTs of psychologically based therapies for chronic NeuP, published in 2015, found only 2 suitable studies.^10^ As individuals with NeuP can have higher degrees of impairment than do individuals with other types of pain,^13–15^ there is clearly a need to determine whether more recent studies of psychologically based treatments for chronic NeuP are available.

Although systematic reviews are essential to inform evidence-based practice,^16^ they are limited in the questions they can answer. In recent years, scoping reviews have been recognized as a broader source of information.^17^^,^^18^ Systematic reviews control for potential risks of bias (typically by excluding smaller and non-RCT studies) to answer a specific and relatively narrow question, such as the efficacy of interventions relative to suitable controls on the outcomes of interest.^10^^,^^17^^,^^19^ In contrast, scoping reviews (which are still conducted in a systematic manner) address broader questions, such as the nature and type of evidence available in the literature.^17^^,^^18^^,^^20^ They can also indicate whether undertaking a systematic review is likely to be meaningful.^18^^,^^19^ Given the outcomes of the most recent systematic reviews for evaluating psychological therapies in adults with chronic NeuP,^10^^,^^11^ a scoping review at this stage would offer the prospect of identifying the volume of potentially relevant studies in the literature and the characteristics of psychologically based interventions studied. This in turn might allow the identification of knowledge gaps in the literature to guide future research efforts.

Accordingly, the aims of this scoping review were (1) to identify the types of psychologically based interventions used to manage pain, pain-related disability, or distress for adults with chronic NeuP; (2) to identify the NeuP conditions included in these studies; (3) to summarize the outcomes reported on measures of pain, disability, and distress; and (4) to determine whether an updated systematic review of these interventions for chronic NeuP was warranted.

## Methods

A preliminary search on September 27, 2021, in Medline and CINAHL found no scoping reviews examining psychologically based interventions for NeuP conditions. Three systematic reviews covering multiple NeuP conditions were found, of which 2 were completed 7 or more years ago^10^^,^^11^ and 1 covered interventions using only 2 databases.^21^

The present scoping review followed methodological guidelines from the JBI Manual for Evidence Synthesis.^19^ The final manuscript was prepared consistent with the Preferred Reporting Items for Systematic Reviews and Meta-Analyses Extension for Scoping Reviews (PRISMA-ScR) guidelines.^20^

### Protocol and registration

The review protocol was prospectively registered with the Open Science Framework on December 6, 2021 (registration DOI: 10.17605/OSF.IO/WNSTM). Two extensions were made to the protocol and are noted in the following sections.

### Eligibility criteria

The target population was adults (≥18 years of age) with NeuP conditions persisting for at least 3 months. Only studies using the current definition of NeuP^1^^,^^2^ were included. To ensure consistency of NeuP conditions, studies were limited to those providing diagnoses conforming to the NeuP criteria used in ICD-11,^22^ or where documented completion of clinical assessments (including a clear history of lesion or disease of the nervous system) was provided, or where the NeuP grading system^23^ was used. Self-report measures alone were considered insufficient to establish the presence of NeuP for this review,^23^ as they are not recommended as standalone diagnostic tools.^24–26^ A broad range of NeuP conditions was considered in the review. To achieve the third and fourth aims of the review, specific focus was given to the quantitative studies (including mixed-methods studies) that reported the outcome measures of interest before and after the intervention. Although an assessment of the quality of the evidence is not normally included in a scoping review,^20^ a sample size of at least 20 in each arm after treatment was used as a guide to assess whether there were sufficient numbers of RCTs to answer the fourth aim of the review. This sample size criterion is typically used in Cochrane systematic reviews.^5^^,^^27^

### Definitions of key criteria

The key criteria for this study were (1) the definition of psychologically based interventions and (2) the outcomes on which they were evaluated.

#### Psychologically based interventions

On the basis of the approach taken in the recent Cochrane systematic review of psychological treatments for chronic pain,^5^ an intervention was considered psychologically based when it consisted of, or integrated, definable psychotherapeutic content derived from an existing psychological model or framework. To be eligible, the psychological treatment needed to be the main intervention of interest in a study and to be facilitated by a qualified health practitioner trained in psychological therapy or supervised by a qualified psychologist. Accordingly, interventions delivered solely online, or using apparatus alone, without therapist involvement were not included in this review.

#### Outcomes of interest

In accordance with the recommendations of Treede et al^3^ for chronic pain severity to be evaluated by reference to pain intensity, distress, and disability, the outcomes of interest were (1) pain intensity, (2) pain-related disability/interference, and (3) distress. To be included in this review, studies needed to report outcome data on pain severity and either pain-related disability/interference or distress.^3^ These outcomes are also consistent with the general expectation that psychologically based treatments would be expected to modify at least disability and distress.^3^ This criterion was added as an extension to the protocol. If no dedicated measure of pain-related disability/interference was present, then the following alternatives were considered: (a) total score or functional subscale of quality-of-life measures (such as SF-36),^28^ (b) functional tests, or (c) measures of sleep disturbance. Likewise, studies were accepted if emotional subscales (or equivalent) of quality-of-life measures were used instead of specific measures of mood/distress. Patient’s global impression of change was also accepted in one study, where it was used in addition to pain intensity. These alternative measures of disability and distress were accepted in order to capture sufficient breadth of evidence in keeping with the nature of a scoping review. Similarly, a composite measure of pain intensity and disability was accepted in one study.^29^

### Context

The review placed no restriction on publication year or published language or on the geographic location, cultural context, and health care setting in which the interventions/studies were carried out.

### Types of studies

Studies that included individuals with non-neuropathic pain conditions in which only aggregate data were reported (as opposed to presenting separate data by pain type) were excluded. However, 3 studies in which less than 10% of the total sample had non-neuropathic pain conditions were accepted (such as studies of individuals with spinal cord injury [SCI]).^30–32^ This criterion was the second extension to the protocol to ensure that the breadth of evidence examined aligns with the purpose of this review. The term *neuropathic pain* was initially introduced in 1994 and was revised in 2011 to eliminate conditions that have since been classified as nociplastic pain.^1^^,^^2^^,^^33–35^ Given the relatively recent revision of the definition, it was considered that inclusion of articles from non–peer-reviewed sources might be misleading because of potential diagnostic confusion. As such, the review focused on papers published in peer-reviewed journals only.

### Information sources

After the preliminary search, a full search (from database inception; using the final search terms) was performed in Medline, Embase, PsycINFO, Cochrane Central Register of Controlled Trials, Scopus, Web of Science, and CINAHL on December 8, 2021, and the search was repeated on February 5, 2023. The search was broadened by searching ClinicalTrials.gov, the Australian New Zealand Clinical Trials Registry (ANZCTR) (performed on December 12, 2021, and on February 5, 2023), and by contacting a selection of field experts to look for additional published papers not identified in earlier searches (from March to April 2022). Where relevant, investigators of the registered RCTs were contacted for published articles. Lastly, the reference list of each study included after the full-text review was reviewed (in May 2022 and in March 2023).

### Search strategy

The search terms were developed in consultation with the research team, external experts in the field, and an experienced librarian. The search strategy combined search terms for NeuP and for psychologically based interventions. The search strategy for psychologically based interventions was adapted from identified systematic reviews in the literature.^5^^,^^10^^,^^36^ The strategy for identifying NeuP was developed by following the ICD-11 classification.^22^^,^^37^^,^^38^ Both search strategies were further refined by reflecting on the text words and the index words identified in the initial limited search. Final database search strategies are presented in the Supplementary Material (S1).

### Study selection

Two reviewers (M.O. and A.A.) independently screened the titles/abstracts and met after the first 20 records had been screened, at the halfway point, and when all the records had been screened to discuss uncertainties about study selection. The same 2 reviewers independently reviewed the full text to determine the eligibility of each study. A reviewer discussion involving the remaining authors (M.K.N., D.S., and P.J.W.) was held to resolve disagreements as required. To assist with this process, M.K.N., D.S., and P.J.W. each reviewed a number of records that required discussion. The reference lists of included papers were screened by M.O. and A.A.

### Data extraction

Identified papers were downloaded into EndNote, and duplicates were removed by the first reviewer (M.O.) following the methodology proposed by Bramer, Giustini, de Jonge, Holland, and Bekhuis.^39^ An extraction table prepared in Microsoft Excel was used for study selection. The data were charted by the following characteristics:

1. author (first);
2. year of publication;
3. NeuP conditions and sample size;
4. study design;
5. psychologically based interventions provided;
6. presence/absence of comparison group and, if present, types of interventions provided; and
7. reported findings on measures of pain, pain-related disability, or distress.

The attrition rate in each study was also examined. However, it was difficult to draw conclusions on this important metric, as only about 60% of the included studies mentioned this information. Thus, attrition rate was not included in the data synthesis. The extraction table/spreadsheet was trialed with the first 20 papers by the 2 reviewers (M.O. and A.A.) and updated throughout the review as required. The data extraction was conducted independently by M.O. and A.A.

### Synthesis of results

The main findings were summarized and discussed in relation to the research questions in accordance with the PRSIMA-ScR guidelines.^20^ The results were summarized in writing and presented in a figure and tables according to the objectives of the review.

## Results

### Selection of sources of evidence

**Figure 1.**
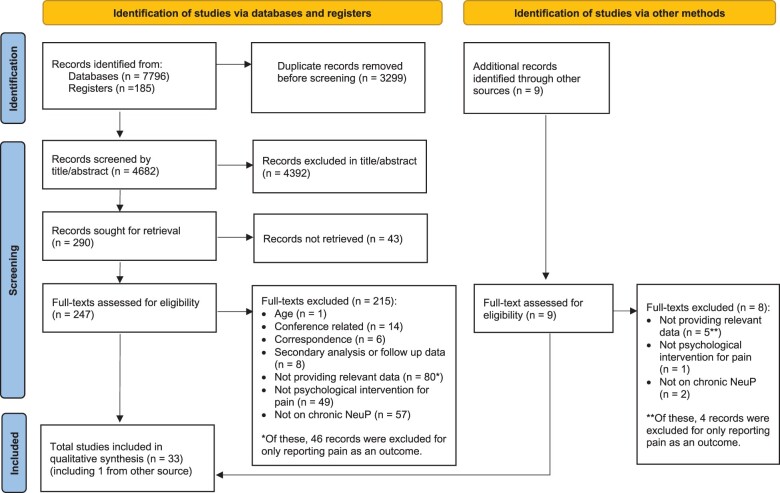
PRISMA flow diagram.

A total of 7981 records were identified through database searching (*n* = 7796) and registers (*n* = 185). These were reduced to 4682 records after removal of duplicates. Of these, 4392 records were excluded in title and abstract screening, and 290 full-text articles were sought for retrieval. After this, 247 full-text articles were obtained and assessed for eligibility. A total of 215 records were excluded for the following reasons: 1 was not on an adult population, 14 were conference abstracts or posters, 6 were letters or correspondence, 8 were secondary analyses or follow-up data of other identified studies, 80 did not provide relevant outcomes of interest (of these, 46 included only pain intensity as an outcome of interest), 49 did not meet the criteria of a psychologically based intervention for pain management, and 57 were not focused on chronic NeuP. After these exclusions, 32 records were identified as eligible. Citation searching of these studies and contacting field experts resulted in the identification of 9 additional articles for eligibility assessment. Of these, 8 were excluded in the eligibility assessment: 5 for not providing relevant outcomes of interest (of these, 4 reported only pain as an outcome), 1 for not meeting the criteria of a psychologically based intervention, and 2 for not focusing on chronic NeuP. After these exclusions, 1 additional eligible paper was identified. In sum, 33 studies met the eligibility criteria and were included in data synthesis. The study selection process is presented in a study selection flow chart (Figure 1).^20^^,^^40^

### Characteristics of individual sources of evidence

The characteristics of psychologically based interventions (intervention type and duration, delivery format, follow-up intervals, and facilitators) in the included studies are summarized in Table 1, along with the first author, year of publication, and NeuP type. The interventions were grouped broadly by the main therapeutic framework/approach. A summary of study designs and key findings of the included studies are presented in Table 2. To allow for cross-table comparisons, data in both tables are presented in the same order by intervention type and study design. For instance, for cognitive-behavioral therapy (CBT)–based pain management programs (PMPs), RCTs are presented first, followed by controlled pre–post studies, uncontrolled pre–post studies, mixed-methods studies, and single-subject designs.

**Table 1.** Summary of psychologically based intervention characteristics (grouped by intervention type).

First author (year)	Intervention type	NeuP type	Delivery format	Duration	Follow-up intervals	Facilitators
Intervention based on cognitive-behavioral approaches (eg, CBT, ACT, BT)						
Heutink (2012)^12^	CBT-based PMP	SCI	F2F group	3 h/wk for 10 weeks, followed by 1 session at 3 weeks after.	6 months.	Psychologist and PT or nurse practitioner and PT.
Norrbrink (2006)^47^	CBT-based PMP	SCI	F2F group	2× weekly for 10 weeks (5 h/wk).	3, 6, and 12 months.	Multidisciplinary team (no further information given)
Nicholson Perry (2010)^32^	CBT-based PMP	SCI	F2F group	10 group sessions, 45 hours in total.	1 and 9 months.	Clinical psychologists, PTs, nurses, and doctors.
Boschen (2016)^44^	CBT-based PMP	Multiple	F2F group	2×/wk over 10 weeks.	Nil.	Physician, SW, OT, or nurse. PT/PT assistant led exercise.
Burns (2013)^46^	CBT-based PMP	SCI	F2F group	2.5-hour, biweekly sessions for 10 consecutive weeks.	3 and 12 months.	OT, SW, and PT.
Shaygan (2018)^48^	CBT-based PMP	Multiple	F2F group and indiv.	15 continuous days (inpatient).	3 months.	Neurologists, PTs, OTs, psychotherapists, and SWs.
Burke (2017)^45^	CBT-based PMP	SCI	F2F group and indiv.	3.5 hours, 2×/wk for 5 weeks.	6 months.	Doctor, clinical psychologist, PT, OT, dietician, SW, and nurse.
Daniel (2021)^29^	CBT-based PMP	TN	F2F group	3 h/wk for 6 weeks.	1 and 9 months.	Clinical psychologist and PT; and clinical nurse specialist or consultant.
Proulx (2018)^49^	CBT-based PMP	SCI	F2F group and indiv.	10 weeks (2× 4-hour group sessions, indiv. sessions, and tailored homework).	At 16, 24, and 36 weeks (group) and as needed (indiv.).	Psychiatrist, PT, physical educator, OT, psychologist, and clinical coordinator.
Saxena (2021)^53^	CBT (with pregabalin)	PHN	F2F, unclear if group or indiv.	12 weeks (45- to 60-min supervised sessions 3 d/wk and skill practice at home).	Nil.	Supervisor who completed intensive training on CBT.
Higgins (2020)^50^	CBT	PDPN	F2F indiv.	10 sessions of 60-min/wk, delivered within 14 weeks.	12 and 36 weeks.	Doctoral-level psychologists.
Otis (2013)^51^	CBT	PDPN	F2F indiv.	60 min/wk for 11 weeks.	4 months.	PhD-level clinical psychologist or masters-level therapist.
Pereira (2021)^52^	CBT	PLP	F2F indiv.	11 sessions over 17 weeks.	2 months. (phone).	Doctoral-level trainee with masters’ degree in clinical psychology.
Scott (2021)^42^	ACT (therapist supported online)	HIV	Online and indiv. feedback	12× 45- to 60-min sessions over 8 weeks. Feedback in writing or by phone (10–30 min).	2 and 5 months.	Clinical psychologists (tailored feedback).
Kioskli (2020)^41^	ACT (therapist supported online)	PDPN	Online, indiv. (Skype and in writing)	8 online sessions (avg. 19 minutes each) over 5 weeks. Plus 1× 30- to 45-min Skype session before and after 5 weeks.	Nil.	Master’s-level health psychologist (Skype sessions and written feedback).
van Laake-Geelen (2021)^43^	Exposure in Vivo	PDPN	F2F indiv.	1 hour, 2×/wk for 8 weeks.	6 months.	Physician, psychologist, PT, and OT.

Mindfulness-based intervention						
Nathan (2017)^60^	MBSR	PDPN	F2F group	2.5 h/wk for 8 weeks, including one 6-hour session midway through the course.	3 months.	Trained and experienced (>5 years) MBSR practitioners.
Shergill (2022)^62^	MBSR	Post-cancer	F2F group	2.5 h/wk for 8 weeks with a full-day retreat held halfway through the treatment.	2 weeks and 3 months.	Psychologists and SWs certified in MBSR, and 2 supervised students.
Zhu (2019)^61^	MBSR	PHN	F2F group and home practice	8-week course and practice 45–60 min/d for 6 d/wk.	Nil.	Psychologist trained in MBSR.
Davoudi (2021)^55^	Mindfulness/meditation	PDPN	F2F group	90 min/wk for 12 weeks.	Nil.	Trained psychotherapists.
Meize-Grochowski (2015)^56^	Mindfulness/meditation	PHN	F2F indiv. and home practice	1-hour session, followed by daily meditation practice using compact discs for 6 weeks. Weekly phone reminder to submit diaries.	Nil.	Certified and experienced (>15 years) MBSR instructor.
Teixeira (2010)^57^	Mindfulness/meditation	PDPN	F2F indiv. and home practice	1-hour session, followed by a guided practice using compact disc 5 d/wk for 4 weeks. Weekly call given.	Nil.	Facilitator experienced and trained in mindfulness and diabetes education.
Tavee (2011)^58^	Mindfulness/meditation	MS and PN	F2F group	90-min/wk class for 8 weeks. Daily home practice was encouraged.	Nil.	Buddhist monk.
Brown (2017)^54^	Mindfulness/meditation	Post-stroke	F2F indiv.	25 min/d using compact disc for 12 weeks. Plus 0.5- to 1-hour weekly meeting to discuss practice difficulties.	3 months.	Trainee in master’s in clinical psychology.
Izgu (2020)^59^	Mindfulness vs PMR	PDPN	F2F indiv. and home practice	12 weeks (1× F2F training session, followed by 20 minutes × 12 weeks home practice using audio files).	2 weeks.	3rd author certified and experienced in PMR and meditation.
Hussain (2019)^63^	MBCT vs PMR	PDPN	F2F group	2×/wk for 8 weeks, each session lasted 30 minutes.	12 weeks after.	Trained and experienced meditation instructors.
Hypnosis						
Dorfman (2013)^67^	Hypnosis	HIV	F2F indiv. and home practice	70 min/wk training in self-hypnosis for 3 weeks, followed by self-practice using audio compact disc.	For 7 weeks (3 visits).	Hypnotherapists.
Jensen (2000)^30^	Hypnosis	SCI	F2F indiv.	One session of hypnotic suggestion for relaxation followed by hypnotic suggestion for analgesia.	Nil.	Clinicians “relatively inexperienced” with hypnosis.
de la Vega (2019)^68^	Hypnotic cognitive therapy	SCI	F2F indiv.	8× 90-minute sessions, delivered weekly for sessions 1–4 and monthly for sessions 5–8.	1 year.	Clinical psychologist.

Originated from trauma-focused therapy						
Kip (2016)^31^	ART	Multiple	F2F indiv.	Avg. of 3.1 sessions for a mean duration of 25 days.	1 month.	Mental health professionals proficient in ART.
De Roos (2010)^65^	EMDR	PLP	F2F indiv.	Avg. of 5.9 sessions (ranged from 3 to 10).	3 months and long term.	Senior psychotherapists trained in EMDR.
Brennstuhl (2015)^64^	EMDR	PBS	F2F indiv.	90 min/wk for 9 weeks (case 1) to 12 weeks (case 2).	3 and 6 months.	Psychotherapists.
Russell (2008)^66^	EMDR	PLP	F2F indiv.	5 sessions over 8 weeks.	3 weeks.	Navy clinical psychologist.

*Abbreviations:*  Intervention types—BT = behavior therapy; ACT= acceptance and commitment therapy; ART= accelerated resolution therapy; CBT= cognitive-behavioral therapy; EMDR= eye movement desensitization and reprocessing; MBCT= mindfulness-based cognitive therapy; MBSR= mindfulness-based stress reduction; PMP= pain management program; PMR= progressive muscle relaxation. Neuropathic pain (NeuP) types—HIV= painful peripheral neuropathy in HIV; MS= multiple sclerosis; multiple= study with multiple NeuP conditions; PN= peripheral neuropathy; PBS= phantom breast syndrome; PDPN= painful diabetic peripheral neuropathy; PHN= postherpetic neuralgia; PLP= phantom limb pain; post-cancer= NeuP in cancer survivors; post-stroke= central poststroke pain; SCI= NeuP associated with spinal cord injury; TN= trigeminal neuralgia. Delivery formats—F2F = face to face; indiv. = individual. Facilitator types—PT= physiotherapist; SW= social worker; OT= occupational therapist.

**Table 2.** Study design and key findings of included studies (listed in the same order as Table 1).

First author (year)	Study design and sample size (analyzed)	Outcome of interest (pain, disability, and distress)	Results / key findings (relating to the outcome of interest)
Heutink (2012)^12^	RCT^a^ *n* = 61 CBT (*n* = 31) or wait-list control (*n* = 30).	**Pain**: CPG **Disability**: CPG **Distress**: HADS (anxiety and depression)	Pain intensity and disability significantly reduced only in the CBT group from pretreatment to posttreatment (*P* < .05), but not from pretreatment to 6 months. Anxiety improved significantly only in the CBT group from pretreatment to posttreatment, and from pretreatment to 6 months (*P* < .05). Neither CBT nor control group showed any improvement in depression.
Norrbrink (2006)^47^	Controlled pre–post study *n* = 38 Intervention (*n* = 27) or control (*n* = 11).	**Pain**: The Borg CR10 **Disability**: Nil, but measured quality of sleep (a 10-item “sleep questionnaire”). **Distress**: The Borg CR10 (unpleasantness) and HADS (anxiety and depression)	There was no change from baseline to 12 months’ follow-up for pain intensity and pain unpleasantness. A systematic reduction in levels of anxiety and depression was found in the treatment group from baseline to the 12 months’ follow-up (95% CI was used). A trend toward improved quality of sleep was also noted. However, only the reduction in depression was significantly different when compared with the control group.
Nicholson Perry (2010)^32^	Controlled pre–post study *n* = 36 PMP (*n* = 19), and Usual care (*n* = 17). (Included as only 8% had non-neuropathic pain).	**Pain**: NRS **Disability**: MPI-SCI (life interference subscale). **Distress**: HADS (anxiety and depression).	The PMP group showed a significantly greater reduction in life interference (*P* < .05) after the treatment, compared with the usual care group. Within the PMP group, a significant reduction in anxiety over time (*P* < .001) and a trend toward significant improvement on pain and depression over time were found. However, these scores did not significantly differ between groups.
Boschen (2016)^44^	Uncontrolled pre–post study NeuP (*n* = 27), along with other pain types (total *n* = 214).	**Pain**: VAS and SF-MPQ **Disability**: PDI **Distress**: BDI-II (depression) and BAI (anxiety)	Overall, significant pretreatment to posttreatment improvements were found in pain, depression, anxiety, and disability (*P* < .01). These outcomes did not differ by pain condition (4 conditions, including widespread pain; neck and back pain; NeuP; and arthritis).
Burns (2013)^46^	Uncontrolled pre–post study *n* = 17	**Pain, disability,** and **distress** measured by MPI-SCI (pain severity, life interference, affective distress).	Pain severity did not change from pretreatment to posttreatment, or at 12 months. A significant change for life interference was found at 12 months (*P* = .003) (not at posttreatment or 3 months). No significant change was found for affective distress or for other measures (except for life control at posttreatment).
Shaygan (2018)^48^	Uncontrolled pre–post study *n* = 141	**Pain**: NRS **Disability**: PDI **Distress**: CESD (depression)	Pain intensity, disability, and depression improved significantly from pretreatment to posttreatment (*P* = .000). These gains were maintained at 3 months, except for depression.
Burke (2017)^45^	Mixed methods (including uncontrolled pre–post study) (Feasibility study) *n* = 8	**Pain**: NRS **Disability**: 6 pain interference items from ISCIPBDS. **Distress**: HADS (depression and anxiety)	A significant reduction in depressive symptoms were found from baseline to posttreatment, and from baseline to 6 months’ follow-up (*P* = .01). No significant improvements were found for pain intensity, pain interference, or anxiety.
Daniel (2021)^29^	Mixed methods (including uncontrolled pre–post study) (Feasibility study) *n* = 15	**Pain**: GCPS* **Disability**: GCPS* **Distress**: DAPOS (depression and anxiety) *Composite measure.	GCPS scores showed a trend for reduction in pain-related disability at 9 months’ follow-up. The study does not provide outcome information specifically for pain intensity. There was a trend for improvement for depression between pretreatment and the 1-month follow-up, and between pretreatment and the 9-month follow-up. No change was found on anxiety.
Proulx (2023)^49^	Single subject design (Pilot study) *n* = 6	**Pain**: NRS **Disability**: BPI **Distress**: HADS (depression and anxiety)	Visual analysis showed significant reductions in pain intensity and pain interference were found in 1 participant and in 5 participants, respectively. There was a significant decrease in anxiety in 5 participants and in depression in 4 participants (visual analysis).
Saxena (2021)^53^	RCT^a^ (Pilot study) *n* = 40 CBT and pregabalin (*n* = 20) or standard care (pregabalin only) (*n* = 20)	**Pain**: NRS and NPSI (symptom severity) **Disability**: Nil, but NRS (sleep interference) and SF-12 (physical health) were measured. **Distress**: BDI-II (depression) and SF-12 (mental health)	A significant group difference in pain (NRS) was found at the end of 8th and 12th weeks (*P* < .01). A greater proportion of participants in the CBT group reported a reduction in pain by the 8th week of the program. At the posttreatment, burning sensation, allodynia, depression, and QoL (physical health component) improved significantly in the CBT group (*P* < .05). No significant group difference was found for mental health component of QoL or sleep interference.
Higgins (2020)^50^	RCT^a^ *n* = 42 CBT (*n* = 21) or Control (diabetic education) (*n* = 21). All received pharmaceutical care.	**Pain**: NRS and NPS (NeuP intensity) **Disability**: MPI-I (the interference subscale) and SF-36 (physical health) **Distress**: BDI (depression) and SF-36 (mental health)	Pain intensity (NRS) significantly reduced in the intervention group at 12 weeks (*P* < .05), but mean reduction in pain intensity did not differ between groups. There was a greater reduction in NeuP intensity in the CBT group at 12 weeks (not at 36 weeks) than in the control group (*P* < .05). No significant group difference in mean change was found for depression (at 12 and 36 weeks). Mental health improved significantly in the CBT group at 36 weeks (not at 12 weeks), compared with the control group (*P* = .01). At 36 weeks, there was significant improvement in pain interference in the CBT group (*P* = .03), but this is largely due to increased pain interference reported in the control group.
Otis (2013)^51^	RCT (Pilot study) *n* = 16 CBT (*n* = 8) or treatment as usual (*n* = 8)	**Pain**: WHYMPI **Disability**: WHYMPI (interference) **Distress**: BDI	A significant decrease in pain (*P* < .01) and pain interference (*P* < .05) was found from pretreatment to 4 months’ follow-up only in the CBT group. There was no significant change in depressive symptoms from pretreatment to 4 months’ follow-up in either condition.
Pereira (2021)^52^	Case study *n* = 1	**Pain**: (PRS) (0–10) **Disability**: POQ (overall impairment) **Distress**: SUDS (distress)	Pain intensity and overall impairment decreased progressively over time, but not distress.
Scott (2021)^42^	RCT (Feasibility trial) *n* = 31 ACT (*n* = 19) or Wait-list control (*n* = 12)	**Pain**: BPI **Disability**: BPI **Distress**: PHQ-9 (depression)	Treatment effect sizes for pain, pain interference, and depression favored the intervention group and were small to moderate at both follow-ups.
Kioskli (2020)^41^	Uncontrolled pre–post study (Feasibility study) *n* = 30	**Pain**: NRS **Disability**: WSAS (functional impairment) **Distress**: NRS (pain distress) and PHQ-9 (depression)	Significant improvements for pain intensity, pain distress, depression, and functional impairment were found in patients who completed the treatment (*P* = 0.000).
van Laake-Geelen (2021)^43^	Single subject design (Pilot study) *n* = 3	**Pain**: VAS (daily pain intensity) **Disability**: PDI **Distress**: HADS (depression and anxiety)	No clear trends were found for pain, depression, or anxiety among 3 participants. A small non-significant reduction in disability was found in all 3 participants from the beginning to the end of the exposure treatment.
Nathan (2017)^60^	RCT^a^ *n* = 62 MBSR (*n* = 30) or wait-list control (*n* = 32)	**Pain**: BPI **Disability**: BPI **Distress**: PHQ-9 (depression), POMS-2A (total mood disturbance), and PSS (perceived stress).	A significantly greater portion of participants in the MBSR group reported a reduction in pain interference from baseline to posttreatment, compared with the control group (*P* < .05). The levels of pain intensity (*P* < .001), depression (*P* < .001), and perceived stress (*P* = 0.001) improved significantly in the MBSR group during the same period, compared with control. The scores on the total mood disturbance decreased in both groups with no significant group difference.
Shergill (2022)^62^	RCT^a^ *n* = 98 MBSR (*n* = 49) or Wait-list control (*n* = 49)	**Pain**: NPSI (total score) **Disability**: BPI **Distress**: PHQ-9 (depression), POMS (mood state), and PSS (perceived stress)	No significant group difference from pretreatment to 3 months’ follow-up was found across all measures.
Zhu (2019)^61^	RCT^a^ *n* = 50 MBSR (*n* = 25) or usual care (*n* = 25)	**Pain**: NPRS **Disability**: Nil **Distress**: HAMD (depression) and HAMA (anxiety)	A significant reduction in pain intensity, depression, and anxiety was found after the MBSR (*P* < .01), but not in the control group.
Davoudi (2021)^55^	RCT^a^ (5 groups) *n* = 204 Mindfulness and placebo (*n* = 40); placebo (*n* = 40); mindfulness (*n* = 41); vitamin D (*n* = 40), or mindfulness and vitamin D (*n* = 43)	**Pain**: NPS (NeuP severity) **Disability**: PDI **Distress**: Nil	Pain severity and pain-related disability significantly improved from pretreatment to posttreatment across all the groups (*P* < .05), except for the placebo-only group. A greater improvement was found in a combined mindfulness and vitamin D group, compared with mindfulness or vitamin D alone (*P* < .05).
Meize-Grochowski (2015)^56^	RCT (Pilot study) *n* = 27 Meditation (*n* = 13) or wait-list control (*n* = 14)	**Pain**: NRS (daily pain level) and SF-MPQ-2 (pain intensity) **Disability**: Nil, but used RAND/SF-36 (physical functioning) **Distress**: CESD (depression), STAI (anxiety), and SF-36 (emotional wellbeing)	Reports improved pain scores in the meditation group at the 0.10 level after the treatment. Physical functioning (SF-36) showed a non-significant increase in the meditation group compared with the control group. Level of anxiety significantly decreased from pretreatment (W2) to posttreatment (W8) for both groups (*P* < .05). Level of depression improved significantly only in the control group from pretreatment to posttreatment (W8) (*P* < .05). Emotional well-being improved slightly in the meditation group.
Teixeira (2010)^57^	RCT (Pilot study) *n* = 20 Meditation (*n* = 10) or attentional control (*n* = 10) (nutritional information and daily food diary).	**Pain**: NPS (Item 1) **Disability**: Nil, but used NeuroQoL **Distress**: NPS (Item 9—pain unpleasantness)	Pain intensity and pain unpleasantness did not differ significantly between groups after controlling for the baseline scores. There was no significant group difference across all the scales of NeuroQoL at posttreatment.
Tavee (2011)^58^	Controlled pre–post study (Pilot study) *n* = 40 Meditation (*n* = 22) or Usual care (*n* = 18)	**Pain**: VAS **Disability**: Nil, but used SF-36 (physical health). **Distress**: Nil, but used SF-36 (mental health)	After 8 weeks, a significant improvement in pain, and SF-36 scores for overall physical health, mental health, vitality, and physical role was found only in the intervention group (all *P* < .05, except for physical role and vitality *P* < .01). MS patients in the intervention group also reported a significant reduction in SF-36 bodily pain scores after the treatment (*P* < .05).
Brown (2017)^54^	Case study *n* = 1	**Pain**: NRS and SF-MPQ-2 (pain intensity and quality) **Disability**: Nil, but used SF-36 (overall physical health) **Distress**: DASS (depression, anxiety and stress) and SF-36 (mental health)	A clinically meaningful pain reduction immediately after mindfulness practice was found (NRS) (*P* < .05). A non-significant reduction in pain from pretreatment to posttreatment on 3 of 4 subscales of SF-MPQ-2 was found, but this gain was not maintained at follow-up. There was a slight decline in physical health from pretreatment to posttreatment. This improved slightly at follow-up. There was a slight improvement in mental health from pretreatment to posttreatment, but this declined at follow-up. On DASS-21, depressive and stress symptoms improved from pretreatment to posttreatment, but the scores worsened at follow-up, compared with the baseline. The score on an anxiety subscale increased from pretreatment to posttreatment, which improved slightly from posttreatment to follow-up.
Izgu (2020)^59^	RCT^a^ (2 interventions and 1 control) *n* = 65 PMR (*n* = 23); meditation (*n* = 21); or attention control (education) (*n* = 21)	**Pain**: VAS **Disability**: Nil, but has data on NEPIQOL **Distress**: Nil	Pain severity scores were significantly lower only in the intervention groups at posttreatment (*P* < .05) and were statistically significant only in the PMR group at the 2-week follow-up. There was no significant group difference for QoL at posttreatment or follow-up. However, in the PMR group, QoL scores were significantly higher after treatment compared with baseline and at follow-up (*P* < .05).
Hussain (2019)^63^	RCT^a^ (2 interventions and 1 control) *n* = 105 MBCT (*n* = 36), PMR (*n* = 32), or control meditation (*n* = 37)	**Pain**: BPI-DPN Q4 (ave. daily pain) **Disability**: Nil, but measured PGIC (impression of change) **Distress**: Nil	Avg. daily pain significantly reduced from pretreatment to posttreatment in both the MBCT and PMR groups (*P* < .05). The reduction in pain was greater for MBCT than for the control group (*P* < .01). PGIC showed a significant improvement in both MBCT (*P* = .01) and PMR groups (*P* = .04), but not in the control group.
Dorfman (2013)^67^	Uncontrolled pre–post study *n* = 36	**Pain**: SF-MPQ (total pain score, VAS, and the present pain intensity) **Disability**: Nil, but used MOS-QOL. **Distress**: CESD (depression) and STAI (anxiety)	The study found significant improvements in pain and QoL (composite score and 4 subscales, including physical functioning) (*P* < .05). A significant decrease in depressive symptoms was also found for those who had elevated scores at baseline (*P* < .05). These gains were maintained for the 7 weeks posttreatment and occurred regardless of participants’ use of medication. There was no change in anxiety associated with the treatment.
Jensen (2000)^30^	Uncontrolled pre–post study *n* = 22 (Included as only 9% had non-neuropathic pain)	**Pain**: NRS (intensity) **Disability**: Nil **Distress**: NRS (pain unpleasantness)	A majority (86%) of participants reported a reduction in pain intensity and pain unpleasantness from pre- to post–hypnotic induction. A significant decrease in pain and pain unpleasantness from pre- to post-induction and an additional significant decrease in pain intensity after the analgesia suggestion were found (*P* < .05).
de la Vega (2019)^68^	Case study *n* = 1	**Pain**: NRS **Disability**: BPI **Distress**: PHQ-8 (depression)	Pain intensity and pain interference showed a clinically meaningful decrease from pretreatment to posttreatment, and these gains were maintained at 1-year follow-up. Depressive symptoms also decreased from pretreatment to posttreatment, but this reduction was not clinically meaningful.
Kip (2016)^31^	Uncontrolled pre–post study (Pilot study) *n* = 10 (Included as only 10% had non-neuropathic pain)	**Pain**: POQ (key domain of pain including pain severity) **Disability**: Nil, but used SF-36 (physical health) **Distress**: CESD (depression), SUDS (distress), and SF-36 (mental health)	Pain severity (POQ) reduced non-significantly from pretreatment to posttreatment and significantly from pretreatment to the 1-month follow-up (*P* < .05). There was no significant change in depression. On SF-36, only bodily pain showed a significant change from pretreatment to posttreatment (*P* < .05), but not from pretreatment to 1 month. The mean SUDS scores reduced significantly from the beginning to the end of session (*P* < .0001).
De Roos (2010)^65^	Uncontrolled pre–post study (Pilot study) *n* = 10	**Pain**: NRS **Disability**: Nil, but used SF-36 (physical functioning) **Distress**: SCL-90 (distress) and SF-36 (mental health). Also used IES and SIL for PTSD symptoms.	Overall, a significant reduction from pretreatment to posttreatment was found for pain intensity (*P* = .00) and distress (total score) (*P* < .05). These gains were maintained at the 3-month follow-up. On SF-36, only vitality and bodily pain showed a significant change over time, which was maintained at follow-up (*P* = .01). There were time interactions between 3 time points of assessment (pretreatment, posttreatment, and follow-up) for total scores of IES and SIL.
Brennstuhl (2015)^64^	Case study *n* = 2	**Pain**: NRS **Disability**: Nil **Distress**: CESD (depression) and STAI (anxiety)	A reduction in pain, anxiety, and depression were reported after an avg. of 10 sessions. One of the patients continued to improve on depression and anxiety throughout the follow-ups with a slight increase in pain intensity at 6 months. The second patient maintained her gains throughout the follow-ups.
Russell (2008)^66^	Case study *n* = 1	**Pain**: NRS **Disability**: Nil **Distress**: BDI (depression), SUDS (distress) and IES-R (PTSD symptoms)	Patient reported an elimination of pain and a reduction in distress, depression, and symptoms of PTSD after 4 sessions of EMDR.

*Abbreviations:*  Pain intensity—BPI= Brief Pain Inventory; BPI-DPN Q4= BPI for painful diabetic peripheral neuropathy; The Borg CR10 scale; CPG= the Chronic Pain Grade questionnaire; GCPS= the Graded chronic pain scale; NRS= Numerical Rating Scale; NPS= the Neuropathic Pain Scale; NPRS= the Numerical Pain Rating Scale; NPSI= Neuropathic Pain Symptom Inventory; POQ= Pain Outcomes Questionnaire; PRS= the Pain Rating Scale; SF-MPQ/SF-MPQ-2= the Short-Form McGill Pain Questionnaire; VAS= visual analogue scale. Disability/functional impairment—BPI= Brief Pain Inventory; CPG= the Chronic Pain Grade questionnaire; GCPS= the Graded chronic pain scale; ISCIPBDS= The International Spinal Cord Injury Pain Basic Dataset, V1; NPS= the Neuropathic Pain Scale—item 9, pain unpleasantness; PDI= the Pain disability index; PGIC= Patients’ Global Impression of Change; POQ= Pain Outcomes Questionnaire; WHYMPI/MPI-I= the West Haven-Yale Multidimensional Pain Inventory; MPI-SCI= the modified spinal cord injury version of MPI; WSAS= Work and Social Adjustment Scale. Distress/mood—BAI= the Beck Anxiety Inventory; BDI/BDI-II= the Beck Depression Inventory; The Borg CR10 scale= pain unpleasantness; CESD= the Center for Epidemiological Studies Depression Scale; DAPOS= the Depression Anxiety and Positive Outlook Scale; DASS-21= the Depression Anxiety Stress Scale; HADS= Hospital Anxiety and Depression Scale; HAMA= The Hamilton Anxiety Rating Scale; HAMD= The Hamilton Depression Rating Scale; PHQ-8/9= Patient Health Questionnaire–8 or 9; POMS-2A= the Profile of Mood States-2A; PSS= the Perceived Stress Scale; SCL-90= the Symptom Checklist 90; STAI= the State-Trait Anxiety Inventory; SUDS= subjective units of distress. Quality of life—NEPIQOL= Neuropathic Pain Impact of Quality of Life; NeuroQoL= the Neuropathy Specific Quality of Life; MOS-QOL= Medical Outcomes Study Quality of Life Measure for HIV-Infected Patients, an adaptation of SF-36; SF-12= Short Form Health Survey-12; SF-36= Short Form Health Survey-36. Other—IES/IES-R= the Impact of Events Scale; SIL= the Self-Inventory List.

a Indicates randomized controlled trials with a sample size of at least 20 in each arm at posttreatment (minimum requirement for systematic review).

### Nature of psychologically based interventions

As presented in Table 1, of the 33 studies included, approximately half of the interventions (48%, *n* = 16) were based on broadly defined cognitive-behavioral approaches. These comprised interventions that were described as using CBT methods (39%, *n* = 13), acceptance and commitment therapy (ACT) methods (6%, *n* = 2),^41^^,^^42^ and exposure in vivo (a common CBT technique that involves systematically encouraging participants to engage in their feared/avoided activities to modify their fears and fearful cognitions and thereby to reduce their level of disability) (3%, *n* = 1).^43^ Nine (27%) of the CBT studies were CBT-based group PMPs facilitated by an interdisciplinary team.^12^^,^^29^^,^^32^^,^^44–49^ The remaining 4 studies consisted of 3 individually based CBT interventions (9%) provided by psychologists alone^50–52^ and 1 CBT intervention (3%) led by a facilitator trained in CBT methods.^53^ Both of the ACT studies were online-based treatments, where online resources were used to provide education and introduce techniques,^41^^,^^42^ but psychologists provided individual input (via means of Skype sessions or tailored feedback) to improve therapy engagement and learning and to address potential therapy-interfering issues. The exposure in vivo intervention was provided on an individual basis by an interdisciplinary team.^43^ Ten (30%) studies examined interventions that used mindfulness/meditation techniques facilitated by certified practitioners, a Buddhist monk, or psychologists. These interventions consisted of studies that used mindfulness/meditation techniques as a standalone intervention individually or in groups (18%, *n* = 6),^54–59^ group-based mindfulness-based stress reduction therapy (9%, *n* = 3),^60–62^ or group-based mindfulness-based cognitive therapy (3%, *n* = 1).^63^ Two (6%) of these studies also included progressive muscle relaxation to examine the relative efficacy of this intervention with mindfulness-based cognitive therapy and mindfulness meditation.^59^^,^^63^ Four (12%) of the studies used individual interventions based on trauma-focused therapy that were facilitated by mental health practitioners. These included 3 (9%) studies that implemented eye movement desensitization and reprocessing^64–66^ and 1 (3%) that used accelerated resolution therapy.^31^ Lastly, 3 (9%) studies used hypnosis (including 1 that used hypnotic cognitive therapy),^30^^,^^67^^,^^68^ facilitated by hypnotherapists or a psychologist.

### NeuP conditions

A range of NeuP conditions were studied, but overall, painful diabetic peripheral neuropathy was the most common (27%, *n* = 9),^41^^,^^43^^,^^50^^,^^51^^,^^55^^,^^57^^,^^59^^,^^60^^,^^63^ closely followed by NeuP associated with SCI (24%, *n* = 8).^12^^,^^30^^,^^32^^,^^45–47^^,^^49^^,^^68^ Four (12%) studies included individuals with phantom limb/breast pain.^52^^,^^64–66^ There were 3 (9%) studies each for multiple NeuP conditions^31^^,^^44^^,^^48^ and for postherpetic neuralgia.^53^^,^^56^^,^^61^ Two (6%) studies were for painful peripheral neuropathy in HIV.^42^^,^^67^ One (3%) study examined individuals with multiple sclerosis and peripheral neuropathy,^58^ and another study was on NeuP in cancer survivors (3%).^62^ The remaining 2 studies included individuals with central poststroke pain (3%)^54^ and with trigeminal neuralgia (3%).^29^

### Study designs

As presented in Table 2, RCTs were the most frequently used study design (*n* = 13, 39% of trials), followed by 8 (24%) uncontrolled, pre–post studies. There were 5 (15%) case studies, 3 (9%) studies with a controlled pre–post study design, and 2 (6%) studies with mixed methods. Both studies with mixed methods used an uncontrolled pre–post study design. The remaining 2 (6%) studies consisted of a single-subject design. Of the 13 RCTs, 9 (69%) trials retained at least 20 participants per study arm at posttreatment (minimum requirement for systematic review). Of the 33 studies, 4 (12%) were described as feasibility studies, and 9 (27%) were described as pilot studies.

### Key outcomes by intervention type

A summary of outcomes by intervention type is provided below. The study designs used in each intervention category are also briefly discussed. As detailed analysis of the outcomes or a statistical comparison of treatment effects was not the purpose of the review, the outcomes are summarized for each intervention category regardless of the study designs used. Follow-up periods are also noted. To summarize the degrees of change after treatments, the results were grouped as “significant” where the studies reported statistically significant results (including 1 study that referred to the clinically meaningful difference scores^68^). The term “mixed” was used to refer to the studies that reported some significant but inconsistent results—for instance, (a) inconsistent results between the different measures of the same/similar dimension (eg, significant for depression but not for anxiety or the “mental health component” of SF-36), or (b) where significant results were reported only at later assessment time points and not immediately after the treatment (eg, significant at 12-month follow-up but not at previous assessment points). Most of the mixed results fell under the reason (a) above. Additionally, 1 study that used a single-subject design was accepted to have mixed results, as the significant results were found in some but not all of the participants in the study.^49^ In contrast, if the studies reported significant outcomes immediately at posttreatment but not at subsequent follow-ups (ie, no maintenance), the results were still classified as “significant.” This is because it is expected that the treatment effects are likely to be at the strongest immediately after the treatment, and it is not clear whether the sudden improvement at later points could be attributed to the treatment. For RCTs and controlled pre–post studies, the results were considered “significant” or “mixed” only if the significant group differences were found in the direction expected. Detailed outcomes of the included studies and their study designs are summarized in Table 2.

#### Cognitive-behavioral approaches

Of the 16 studies identified, 5 (31%) used RCT designs, 2 (13%) used controlled pre–post designs, 6 (38%) used uncontrolled pre–post designs, 2 (13%) used a single-subject design, and 1 (6%) used a case study design. Within these approaches, 15 (94%) studies reported outcomes on pain (as stated earlier, 1 study used a composite pain and disability score), and 16 (100%) studies reported outcomes on disability and distress. Significant results are reported in 6 (40%) of the studies that reported on pain,^12^^,^^41^^,^^44^^,^^48^^,^^51^^,^^53^ 6 (38%) of the studies that assessed disability,^12^^,^^32^^,^^41^^,^^44^^,^^48^^,^^51^ and 3 (19%) of the studies that measured distress.^41^^,^^44^^,^^48^ Additionally, some significant but mixed results are reported in 2 (13%) of the studies that assessed pain, ^49^^,^^50^ 3 (19%) of the studies that measured disability, ^46^^,^^49^^,^^53^ and 6 (38%) of the studies that examined distress.^12^^,^^45^^,^^47^^,^^49^^,^^50^^,^^53^

#### Mindfulness-based intervention

Of the 10 studies identified in this category, 8 (80%) were conducted as RCTs, 1 (10%) used a controlled pre–post study design, and 1 (10%) used a case study design. Of the 10 studies, all assessed pain, 9 (90%) also assessed disability, and 7 (70%) also assessed distress. Significant improvements are reported in 6 (60%) of the studies that assessed pain,^55^^,^^58–61^^,^^63^ 4 (44%) of the 9 studies that measured disability,^55^^,^^58^^,^^60^^,^^63^ and 2 (29%) of the 7 studies that assessed distress.^58^^,^^61^ Additionally, some significant but mixed results were found in 1 (10%) of the studies that measured pain^54^ and 1 (14%) of the 7 studies that assessed distress.^60^ None of the studies that examined disability reported mixed results.

#### Hypnosis

Of the 3 studies that used hypnosis, 2 (67%) used uncontrolled, pre–post designs, and 1 (33%) used a case study design. Of these 3 studies, all measured pain and distress, and 2 (67%) also assessed disability. All 3 studies reported significant improvements in pain intensity.^30^^,^^67^^,^^68^ In terms of disability and distress, significant improvements were reported by 2 (100%) of the 2 studies that measured disability^67^^,^^68^ and 1 (33%) of the studies that assessed distress.^30^ Additionally, 1 study reported mixed results for distress (significant improvements in depression but not in anxiety).^67^

#### Originated from trauma-focused therapy

Of the 4 studies that used interventions derived from trauma-focused therapy, 2 (50%) used an uncontrolled, pre–post design, and the other 2 (50%) were case studies. All 4 studies assessed pain and distress, but only 2 (50%) assessed disability. Significant improvements were found in 1 (25%) of the studies that assessed pain,^65^ and none of the studies that assessed disability or distress reported significant results.^31^^,^^64–66^ Additionally, 1 (25%) of the studies that assessed pain found a significant improvement only from pretreatment to the follow-up (but not from pretreatment to posttreatment).^31^ Two (50%) of the studies that assessed distress reported mixed results.^31^^,^^65^

## Discussion

This review aimed to identify the types of psychologically based interventions studied for the management of pain, pain-related disability, or distress in adults with chronic NeuP. Types of NeuP conditions addressed and the studies’ reported findings were also summarized. After screening and eligibility assessment, 33 studies (with 1453 study participants in total) were identified.

### Psychologically based intervention summary

Four broad intervention approaches were identified. These were interventions based on cognitive-behavioral approaches, mindfulness/meditation, trauma-focused therapy, and hypnosis. CBT, in particular CBT-based PMPs, was the most frequently studied therapeutic approach in adults with chronic NeuP conditions. The prevalence of CBT-based studies is consistent with a recent Cochrane systematic review.^5^ Only the CBT-based PMPs involved an interdisciplinary team. Meditation/mindfulness was the most frequently used individual therapeutic technique, but these were sometimes provided alongside other modalities, such as activity, yoga, or medication. A mindfulness technique was also used in 2 of the CBT-based studies.^29^^,^^47^ Most CBT-based studies included various relaxation methods, but it is unclear whether one was any better than the others^69^ or for which patients. Interventions in approximately half of the studies were delivered individually (including 2 therapist-supported online interventions), followed by group delivery and a combination of group and individual treatment. Analysis of the relative benefits of intervention types was beyond the scope of the present review, and it is an area that could be addressed in a future investigation with a systematic review.

### NeuP conditions by intervention type

The review found that most of the studies examined individuals with a single NeuP condition. Only 3 studies examined multiple NeuP conditions. For CBT-based studies, NeuP associated with SCI was the most frequently studied condition. For studies that included meditation/mindfulness techniques, painful diabetic peripheral neuropathy was the most frequently studied NeuP condition, followed by postherpetic neuralgia. Two of the 3 studies using hypnosis were conducted in individuals with SCIs, whereas 3 of the 4 studies of eye movement desensitization and reprocessing were conducted in individuals with phantom limb/breast conditions. The reason for these differences was unclear, and the choice might well have been based on convenience or ease of access to the participants, rather than a theoretical or mechanism-based analysis of chronic NeuP. Future studies examining psychologically based interventions covering multiple NeuP conditions would be clinically more relevant for most pain clinic settings given their typically heterogeneous patient populations.

### Key findings with regard to the outcome measures

Overall, of the 32 out of 33 studies that reported pain intensity outcomes, 16 (50%) studies reported significant improvements, and 4 (13%) studies reported some significant but mixed results on this dimension. Of the 29 out of 33 studies that assessed disability, 12 (41%) reported significant reductions, and 3 (10%) reported mixed results. Lastly, of the 30 out of 33 studies that measured distress, 6 (20%) reported significant improvements, and 10 (33%) found significant but mixed outcomes. If these results (significant and mixed) are combined, improvements were reported in the following proportions: pain (63%), disability (52%), and distress (53%). However, the results of these studies need to be interpreted with caution because of their study designs, which are less rigorous than would be acceptable for a systematic review, and the limited range of NeuP conditions treated. At present, it is not known whether the limitations in methods and samples treated in these studies would have affected their outcomes or their generalizability.

It should be noted that despite some early claims that people with chronic NeuP experienced more severe pain, distress, and disability than did people with non-neuropathic chronic pain,^13–15^ there is accumulating evidence that the 2 groups share similar psychological characteristics.^8^^,^^9^ Given the evidence supporting the use of psychologically based interventions for adults with chronic pain conditions more broadly,^5^^,^^6^ it is reasonable to expect that psychologically based interventions targeting variables such as pain and disability, as well as pain cognitions and distress, should be equally effective in people diagnosed with chronic NeuP conditions. The findings of this scoping review are consistent with this perspective, but confirmation will require a formal systematic review and meta-analysis of only RCT studies that meet rigorous inclusion criteria.

### RCTs of sufficient sample size

One of the aims of this study was to determine whether there were now enough RCTs of sufficient sample size to warrant undertaking an updated systematic review and meta-analysis. Earlier systematic reviews of psychologically based interventions in adults with NeuP conditions identified only 2–5 studies and concluded that there was insufficient evidence to support these treatments.^10^^,^^11^^,^^21^ The present review found that 13 of the 33 identified studies had RCT study designs. Of these 13 RCTs, 9 trials met the sample size criterion of a minimum of 20 participants per study arm at posttreatment (refer to Table 2). As this represents an improvement over the number of RCTs identified in the earlier systematic reviews mentioned, this suggests it is now time to undertake an updated systematic review and meta-analysis. However, as a risk-of-bias assessment is an essential component of a systematic review^20^^,^^40^^,^^70^ (unlike a scoping review), a proportion of these trials could be unsuitable for a systematic review. Even so, the findings reported here indicate that it would be worth undertaking another systematic review of psychologically based interventions for people with chronic NeuP.

### Future research direction

In full-text review for eligibility, 50 studies (20% of the total assessed) were excluded for not meeting the criteria for a psychologically based intervention. As found by others,^73^ many studies (14 of 50) were excluded because of insufficient information about the treatment providers. It is recommended that future studies of psychologically based interventions provide clear descriptions of the interventions used, why they might be considered “psychologically based,” and the training and experience of the treatment providers. These improvements would assist with future reviews assessing the role of psychologically based interventions in populations with chronic NeuP.

Outcome measures are also a concern. Of the 256 records assessed for eligibility, 50 studies (20%) were excluded as they used only pain (intensity/quality) as an outcome and not pain-related disability or distress. This finding suggests that a considerable proportion of the studies of psychologically based interventions for NeuP did not follow a biopsychosocial perspective of chronic pain, supporting the recent observation that though the biopsychosocial model of pain is widely accepted in principle, it is not always reflected in practice.^71^ It is recommended that future studies of psychologically based interventions of chronic NeuP include dedicated measures of pain-related disability and distress, as well as measures of pain-related cognitions and pain intensity, which would be more consistent with a biopsychosocial view of chronic pain and the ICD-11,^3^^,^^72^ as well as with Initiative on Methods, Measurement, and Pain Assessment in Clinical Trials (IMMPACT) recommendations.^73^

To our knowledge, this is the first scoping review to examine the extent and nature of studies of psychologically based interventions for adults with a wide range of chronic NeuP conditions. The strengths of the review include a comprehensive and rigorous review process involving 2 independent reviewers and the inclusion of pain-related disability or distress as primary outcome data alongside pain intensity. This approach is consistent with a biopsychosocial perspective for understanding and treating individuals with chronic pain.^3^ This review also used an extensive information source, covering 7 databases, along with 2 clinical trials registers, input from field experts, and citation search of the included studies.

### Limitations

Given the nature of a scoping review, risk of bias was not assessed, and non-RCTs were included. Accordingly, key findings with regard to the outcomes of interest are presented as a descriptive summary, and it is not intended as an evaluation of efficacy or effectiveness. However, a limited quality assurance measure was taken by excluding studies that were not published in peer-reviewed journals.

## Conclusions

This scoping review found 4 categories of psychologically based intervention approaches that have been used for adults with chronic NeuP. CBT was the most frequently studied intervention approach. Mindfulness/meditation was the most frequently used technique, but it was sometimes provided alongside other modalities, making it difficult to draw conclusions about its value. Only CBT-based PMPs were conducted by an interdisciplinary team. About 88% of the identified studies focused on a single NeuP condition, with painful diabetic peripheral neuropathy being the most frequently studied NeuP condition, followed by SCI. Approximately one-half to two-thirds of the studies reported improvements in pain (63%), disability (52%), or distress (53%). This indicates that there is a potential role for psychologically based interventions in the management of people seeking help for chronic NeuP conditions. The availability of sufficient RCTs within these studies could be used to confirm this finding through an updated systematic review.

## Supplementary Material

pnae006_Supplementary_Data

## References

[1] Haanpää M , Attal N, Backonja M, et al NeuPSIG guidelines on neuropathic pain assessment. PAIN^®^. 2011;152(1):14-27. 20851519 10.1016/j.pain.2010.07.031

[2] Jensen TS , Baron R, Haanpää M, et al A new definition of neuropathic pain. Pain. 2011;152(10):2204-2205. 21764514 10.1016/j.pain.2011.06.017

[3] Treede R-D , Rief W, Barke A, et al Chronic pain as a symptom or a disease: the IASP Classification of Chronic Pain for the *International Classification of Diseases* (ICD-11). Pain. 2019;160(1):19-27. 30586067 10.1097/j.pain.0000000000001384

[4] Colloca L , Ludman T, Bouhassira D, et al Neuropathic pain. Nat Rev Dis Primers. 2017;3:1-19. 10.1038/nrdp.2017.2 PMC5371025 28205574

[5] Williams AC , Fisher E, Hearn L, Eccleston C; Cochrane Pain, Palliative and Supportive Care Group. Psychological therapies for the management of chronic pain (excluding headache) in adults. Cochrane Database Syst Rev. 2020;(8):1-171. 10.1002/14651858.CD007407.pub4 PMC7437545 32794606

[6] Kamper SJ , Apeldoorn AT, Chiarotto A, et al Multidisciplinary biopsychosocial rehabilitation for chronic low back pain: Cochrane systematic review and meta-analysis. BMJ. 2015;350(5):h444. 10.1136/bmj.h444 25694111 PMC4353283

[7] Day MA , Brinums M, Craig N, et al Predictors of responsivity to interdisciplinary pain management. Pain Med. 2018;19(9):1848-1861. 29025136 10.1093/pm/pnx169

[8] Gustin S , Wilcox S, Peck C, Murray G, Henderson L. Similarity of suffering: equivalence of psychological and psychosocial factors in neuropathic and non-neuropathic orofacial pain patients. Pain. 2011;152(4):825-832. 21316857 10.1016/j.pain.2010.12.033

[9] Daniel HC , Narewska J, Serpell M, Hoggart B, Johnson R, Rice AS. Comparison of psychological and physical function in neuropathic pain and nociceptive pain: implications for cognitive behavioral pain management programs. Eur J Pain. 2008;12(6):731-741. 18164225 10.1016/j.ejpain.2007.11.006

[10] Eccleston C , Hearn L, Williams AC, Cochrane Pain, Palliative and Supportive Care Group. Psychological therapies for the management of chronic neuropathic pain in adults. Cochrane Database Syst Rev. 2015;(10):1-32. 10.1002/14651858.CD011259.pub2 PMC6485637 26513427

[11] van de Wetering EJ , Lemmens KM, Nieboer AP, Huijsman R. Cognitive and behavioral interventions for the management of chronic neuropathic pain in adults—a systematic review. Eur J Pain. 2010;14(7):670-681. 20096614 10.1016/j.ejpain.2009.11.010

[12] Heutink M , Post MWM, Lindeman E, et al The CONECSI trial: results of a randomized controlled trial of a multidisciplinary cognitive behavioral program for coping with chronic neuropathic pain after spinal cord injury. Pain. 2012;153(1):120-128. 10.1016/j.pain.2011.09.029 22100355

[13] Attal N , Lanteri-Minet M, Laurent B, Fermanian J, Bouhassira D. The specific disease burden of neuropathic pain: results of a French nationwide survey. Pain. 2011;152(12):2836-2843. 22019149 10.1016/j.pain.2011.09.014

[14] Inoue S , Taguchi T, Yamashita T, Nakamura M, Ushida T. The prevalence and impact of chronic neuropathic pain on daily and social life: a nationwide study in a Japanese population. Eur J Pain. 2017;21(4):727-737. 28107599 10.1002/ejp.977 PMC5363338

[15] Smith BH , Torrance N, Bennett MI, Lee AJ. Health and quality of life associated with chronic pain of predominantly neuropathic origin in the community. Clin J Pain. 2007;23(2):143-149. 17237663 10.1097/01.ajp.0000210956.31997.89

[16] Pearson A. Balancing the evidence: incorporating the synthesis of qualitative data into systematic reviews. JBI Rep. 2004;2(2):45-64.

[17] Arksey H , O'Malley L. Scoping studies: towards a methodological framework. Int J Soc Res Methodol. 2005;8(1):19-32.

[18] Munn Z , Peters MD, Stern C, Tufanaru C, McArthur A, Aromataris E. Systematic review or scoping review? Guidance for authors when choosing between a systematic or scoping review approach. BMC Med Res Methodol. 2018;18(1):143-147. 30453902 10.1186/s12874-018-0611-x PMC6245623

[19] Peters M , Godfrey C, McInerney P, Munn Z, Tricco A, Khalil H. Scoping reviews. In: Aromataris E, Munn Z, eds. JBI Manual for Evidence Synthesis. JBI; 2020:406-451. https://synthesismanual.jbi.global. 10.11124/JBIES-20-00167 33038124

[20] Tricco AC , Lillie E, Zarin W, et al PRISMA extension for scoping reviews (PRISMA-ScR): Checklist and explanation. Ann Intern Med. 2018;169(7):467-473. 30178033 10.7326/M18-0850

[21] Moisset X , Bouhassira D, Couturier JA, et al Pharmacological and non-pharmacological treatments for neuropathic pain: systematic review and French recommendations. Rev Neurol (Paris). 2020;176(5):325-352. 32276788 10.1016/j.neurol.2020.01.361

[22] Scholz J , Finnerup NB, Attal N, et al Classification Committee of the Neuropathic Pain Special Interest Group (NeuPSIG). The IASP classification of chronic pain for ICD-11: chronic neuropathic pain. Pain. 2019;160(1):53-59. 30586071 10.1097/j.pain.0000000000001365 PMC6310153

[23] Finnerup NB , Haroutounian S, Kamerman P, et al Neuropathic pain: an updated grading system for research and clinical practice. Pain. 2016;157(8):1599-1606. 27115670 10.1097/j.pain.0000000000000492 PMC4949003

[24] Dunker Ø , Grotle M, Kvaløy MB, et al Accuracy of neuropathic pain measurements in patients with symptoms of polyneuropathy: validation of painDETECT, S-LANSS, and DN4. Pain. 2022;164(5):991-1001. 36240023 10.1097/j.pain.0000000000002793

[25] Mathieson S , Maher CG, Terwee CB, De Campos TF, Lin C-WC. Neuropathic pain screening questionnaires have limited measurement properties. A systematic review. J Clin Epidemiol. 2015;68(8):957-966. 25895961 10.1016/j.jclinepi.2015.03.010

[26] Attal N , Bouhassira D, Baron R. Diagnosis and assessment of neuropathic pain through questionnaires. Lancet Neurol. 2018;17(5):456-466. 10.1016/S1474-4422(18)30071-1 29598922

[27] Williams AC , Eccleston C, Morley S. Psychological therapies for the management of chronic pain (excluding headache) in adults. Cochrane Database Syst Rev. 2012;(11):1-100. 10.1002/14651858.CD007407.pub3 PMC6483325 23152245

[28] Ware JE Jr , Sherbourne CD. The MOS 36-item short-form health survey (SF-36): I. Conceptual framework and item selection. Medical Care. 1992;30(6):473-483. 1593914

[29] Daniel HC , Poole JJ, Klein H, Huang C, Zakrzewska JM. Cognitive behavioral therapy for patients with trigeminal neuralgia: a feasibility study. J Oral Facial Pain Headache. 2021;35(1):30-34. 10.11607/ofph.2664 33730124

[30] Jensen MP , Barber J, Williams-Avery RM, Flores L, Brown MZ. The effect of hypnotic suggestion on spinal cord injury pain. BMR. 2000;14(1-2):3-10. 10.3233/BMR-2000-141-202

[31] Kip KE , Tofthagen C, D'Aoust RF, Girling SA, Harper Y, Rosenzweig L. Pilot study of accelerated resolution therapy for treatment of chronic refractory neuropathic pain. Alternat Complement Ther. 2016;22(6):243-250. 10.1089/act.2016.29082.kek

[32] Nicholson Perry K , Nicholas MK, Middleton JW. Comparison of a pain management program with usual care in a pain management center for people with spinal cord injury-related chronic pain. Clin J Pain. 2010;26(3):206-216. 10.1097/AJP.0b013e3181bff8f3 20173434

[33] Merskey H , Bogduk N. Classification of Chronic Pain: Descriptions of Chronic Pain Syndromes and Definitions of Pain Terms. 2nd ed. International Association for the Study of Pain Press; 1994.

[34] Treede R-D , Jensen TS, Campbell JN, et al Neuropathic pain: redefinition and a grading system for clinical and research purposes. Neurology. 2008;70(18):1630-1635. 18003941 10.1212/01.wnl.0000282763.29778.59

[35] Kosek E , Cohen M, Baron R, et al Do we need a third mechanistic descriptor for chronic pain states? Pain. 2016;157(7):1382-1386. 26835783 10.1097/j.pain.0000000000000507

[36] Batho A , Kneale D, Sutcliffe K, Williams AC. Sufficient conditions for effective psychological treatment of chronic pain: a qualitative comparative analysis. Pain. 2021;162(10):2472-2485. 34534175 10.1097/j.pain.0000000000002242

[37] Korwisi B , Hay G, Attal N, et al Classification algorithm for the ICD-11 chronic pain classification (CAL-CP): development and results from a preliminary pilot evaluation. Pain. 2021;162(7):2087-2096. 33492033 10.1097/j.pain.0000000000002208

[38] Nicholas MK , Vlaeyen JW, Rief W, et al; IASP Taskforce for the Classification of Chronic Pain. The IASP classification of chronic pain for ICD-11: chronic primary pain. Pain. 2019;160(1):28-37. 30586068 10.1097/j.pain.0000000000001390

[39] Bramer WM , Giustini D, de Jonge GB, Holland L, Bekhuis T. De-duplication of database search results for systematic reviews in EndNote. J Med Libr Assoc. 2016;104(3):240-243. 27366130 10.3163/1536-5050.104.3.014 PMC4915647

[40] Page MJ , McKenzie JE, Bossuyt PM, et al The PRISMA 2020 statement: an updated guideline for reporting systematic reviews. Syst Rev. 2021;10(1):89-11. 33781348 10.1186/s13643-021-01626-4 PMC8008539

[41] Kioskli K , Godfrey E, Scott W, Winkley K, McCracken LM. Online acceptance and commitment therapy for people with painful diabetic neuropathy in the United Kingdom: a single-arm feasibility trial. Pain Med. 2020;21(11):2777-2788. 10.1093/PM/PNAA110 32358608 PMC7685693

[42] Scott W , Guildford BJ, Badenoch J, et al Feasibility randomized-controlled trial of online acceptance and commitment therapy for painful peripheral neuropathy in people living with HIV: the OPEN study. Eur J Pain. 2021;25(7):1493-1507. 10.1002/ejp.1762 33711209

[43] van Laake-Geelen CC , Smeets RJ, Goossens ME, Verbunt JA. Effectiveness of exposure in vivo for patients with painful diabetic neuropathy: a pilot study of effects on physical activity and quality of life. J Rehabil Med-Clin Commun. 2021;4:1-11. 10.2340/20030711-1000046 PMC8054751 33884148

[44] Boschen KA , Robinson E, Campbell KA, et al Results from 10 years of a CBT pain self-management outpatient program for complex chronic conditions. Pain Res Manag. 2016;2016:4678083. 10.1155/2016/4678083 27891062 PMC5116337

[45] Burke D , Lennon O, Nolan M, et al A cognitive behavioural therapy pain management programme for neuropathic pain post spinal cord injury: a feasibility study including the clinician and patient perspectives. Phys Med Rehabil Int. 2017;4(3):1119-1129.

[46] Burns AS , Delparte JJ, Ballantyne EC, Boschen KA. Evaluation of an interdisciplinary program for chronic pain after spinal cord injury. PM R. 2013;5(10):832-838. 10.1016/j.pmrj.2013.05.004 23684779

[47] Norrbrink Budh C , Kowalski J, Lundeberg T. A comprehensive pain management programme comprising educational, cognitive and behavioural interventions for neuropathic pain following spinal cord injury. J Rehabil Med. 2006;38(3):172-180. 10.1080/16501970500476258 16702084

[48] Shaygan M , Boger A, Kroner-Herwig B. Predicting factors of outcome in multidisciplinary treatment of chronic neuropathic pain. J Pain Res. 2018;11:2433-2443. 10.2147/JPR.S175817 30425557 PMC6204857

[49] Proulx K , Lamontagne ME, Quirion R, Deaudelin I, Mercier C, Perreault K. A six-participant pilot single-subject study of an individualized pain management program for people with spinal cord injury. Spinal Cord Ser Cases. 2023;9(1):2. 10.1038/s41394-022-00557-z PMC9842717 36646690

[50] Higgins DM , LaChappelle KM, Czlapinski R, et al A randomized controlled trial of cognitive behavioral therapy compared with diabetes education for diabetic peripheral neuropathic pain. J Health Psychol. 2020;27(3):649-662. 10.1177/1359105320962262 33070667

[51] Otis JD , Sanderson K, Hardway C, Pincus M, Tun C, Soumekh S. A randomized controlled pilot study of a cognitive-behavioral therapy approach for painful diabetic peripheral neuropathy. J Pain. 2013;14(5):475-482. 23452825 10.1016/j.jpain.2012.12.013

[52] Pereira L , Noronha D, Bishop A. Cognitive behavioral therapy for postamputation chronic pain: a case report. Cognit Behav Pract. 2021;30(1):160-168. 10.1016/j.cbpra.2021.07.002

[53] Saxena AK , Bhardwaj N, Chilkoti GT, et al Modulation of mRNA expression of IL-6 and mTORC1 and efficacy and feasibility of an integrated approach encompassing cognitive behavioral therapy along with pregabalin for management of neuropathic pain in postherpetic neuralgia: a pilot study. Pain Med. 2021;22(10):2276-2282. 10.1093/pm/pnab142 34097069

[54] Brown A , Becerra R. Mindfulness for neuropathic pain: a case study. Health & Mental Health Treatment & Prevention 3300. Int J Psychol Psychol Ther. 2017;17(1):19-37.

[55] Davoudi M , Allame Z, Niya RT, Taheri AA, Ahmadi SM. The synergistic effect of vitamin D supplement and mindfulness training on pain severity, pain-related disability and neuropathy-specific quality of life dimensions in painful diabetic neuropathy: a randomized clinical trial with placebo-controlled. J Diabetes Metab Disord. 2021;20(1):49-58. 10.1007/s40200-020-00700-3 34222059 PMC8212219

[56] Meize-Grochowski R , Shuster G, Boursaw B, et al Mindfulness meditation in older adults with postherpetic neuralgia: a randomized controlled pilot study. Geriatric Nursing (New York, NY). 2015;36(2):154-160. 10.1016/j.gerinurse.2015.02.012 PMC4488325 25784079

[57] Teixeira E. The effect of mindfulness meditation on painful diabetic peripheral neuropathy in adults older than 50 years. Holist Nurs Pract. 2010;24(5):277-283. 20706089 10.1097/HNP.0b013e3181f1add2

[58] Tavee J , Rensel M, Planchon SM, Butler RS, Stone L. Effects of meditation on pain and quality of life in multiple sclerosis and peripheral neuropathy: a pilot study. Int J MS Care. 2011;13(4):163-168. 24453721 10.7224/1537-2073-13.4.163 PMC3882962

[59] Izgu N , Gok Metin Z, Karadas C, Ozdemir L, Metinarikan N, Corapcıoglu D. Progressive muscle relaxation and mindfulness meditation on neuropathic pain, fatigue, and quality of life in patients with type 2 diabetes: a randomized clinical trial. J Nurs Scholarsh. 2020;52(5):476-487. 10.1111/jnu.12580 32536026

[60] Nathan HJ , Poulin P, Wozny D, et al Randomized trial of the effect of mindfulness-based stress reduction on pain-related disability, pain intensity, health-related quality of life, and A1c in patients with painful diabetic peripheral neuropathy. Clin Diabetes. 2017;35(5):294-304. 10.2337/cd17-0077 29263572 PMC5734176

[61] Zhu X , Gao H, Hu P, et al Effects of mindfulness-based stress reduction on depression, anxiety, and pain in patients with postherpetic neuralgia. J Nerv Ment Dis. 2019;207(6):482-486. 10.1097/NMD.0000000000000998 31045954

[62] Shergill Y , Rice DB, Khoo EL, et al Mindfulness-based stress reduction in breast cancer survivors with chronic neuropathic pain: a randomized controlled trial. Pain Res Manag. 2022;2022:1-14. 10.1155/2022/4020550 PMC9282981 35845983

[63] Hussain N , Said ASA. Mindfulness-based meditation versus progressive relaxation meditation: impact on chronic pain in older female patients with diabetic neuropathy. J Evid Based Integr Med. 2019;24:1-8. 10.1177/2515690X19876599 PMC6757487 31544476

[64] Brennstuhl MJ , Tarquinio C, Montel S, Masson J, Bassan F, Tarquinio P. Using eye movement desensitization and reprocessing (EMDR) as a treatment for phantom breast syndrome: case study. Utilisation de la therapie EMDR-eye movement desensitization and reprocessing—pour le traitement du syndrome du sein fantome: etude de cas. Sexologies. 2015;24(2):56-64. 10.1016/j.sexol.2014.09.005

[65] De Roos C , Veenstra AC, De Jongh A, et al Treatment of chronic phantom limb pain using a trauma-focused psychological approach. Pain Res Manag. 2010;15(2):65-71. 10.1155/2010/981634 20458374 PMC2886995

[66] Russell MC. Treating traumatic amputation-related phantom limb pain: a case study utilizing eye movement desensitization and reprocessing within the armed services. Clin Case Stud. 2008;7(2):136-153. 10.1177/1534650107306292

[67] Dorfman D , George MC, Simpson DM, Davidson G, Schnur J, Montgomery G. Hypnosis for treatment of HIV neuropathic pain: a preliminary report. Pain Med. 2013;14(7):1048-1056. 10.1111/pme.12074 23566167

[68] de la Vega R , Mendoza ME, Chan JF, Jensen MP. Case study: cognitive restructuring hypnosis for chronic pain in a quadriplegic patient. Am J Clin Hypn. 2019;61(4):394-408. 10.1080/00029157.2018.1537973 31017549

[69] Dunford E , Thompson M. Relaxation and mindfulness in pain: a review. Rev Pain. 2010;4(1):18-22. 10.1177/204946371000400105 PMC4590063 26524978

[70] Moher D , Liberati A, Tetzlaff J, Altman DG, Group P. Preferred reporting items for systematic reviews and meta-analyses: the PRISMA statement. Ann Intern Med. 2009;151(4):264-269, W64. 19622511 10.7326/0003-4819-151-4-200908180-00135

[71] Nicholas MK. The biopsychosocial model of pain 40 years on: time for a reappraisal? PAIN. 2022;163(suppl 1):S3-S14. 36252231 10.1097/j.pain.0000000000002654

[72] Treede R-D , Rief W, Barke A, et al A classification of chronic pain for ICD-11. Pain. 2015;156(6):1003-1007. 25844555 10.1097/j.pain.0000000000000160 PMC4450869

[73] Turk DC , Dworkin RH, Allen RR, et al Core outcome domains for chronic pain clinical trials: IMMPACT recommendations. Pain. 2003;106(3):337-345. 14659516 10.1016/j.pain.2003.08.001

