# Supplementary material for: Psychologically based interventions for adults with chronic neuropathic pain: a scoping review
Source: Pain Med. 2024 Feb 4;25(6):400–14. doi: 10.1093/pm/pnae006 (PMC11145456; doi:10.1093/pm/pnae006)
Supplement: pnae006_Supplementary_Data [file pnae006_supplementary_data.pdf]

Supplementary material S1.

Section 1. Data source

1. Databases (Searched on 8 December 2021 and on 5 February 2023)

    - Medline (OVID)
    - Embase (OVID)
    - PsycINFO (OVID)
    - Cochrane Central (OVID)
    - CINAHL Complete
    - Scopus
    - Web of science – Core Collections

2. Clinical trial registry searched (Searched on 12 December 2021 and on 5 February 2023)
    - Clinicaltrials.gov
    - Australian New Zealand Clinical Trials Registry (ANZCTR)

3. Other sources

a) Papers identified through the reference list of included papers

    Searched in May 2022 and in March 2023

b) A selection of field experts contacted for possible additional papers (March-April 2022)

    - Dr Carolyn Berryman, School of biomedicine, Faculty of health and Medical Sciences, The University of Adelaide

    - Prof. Andrew Rice, Faculty of medicine, Department of Surgery & Cancer, Imperial College London

    - Prof. Toby Newton John, the University of Technology Sydney

    - Prof. Louise Sharp, the University of Sydney

    - Prof. Amanda Williams, the University College London

    - Prof. Philip Siddall, HammondCare

    - Prof. Ashley Craig, the University of Sydney

    - Dr Janet Bultitude (the University of Bath)

    - Prof. Mark P. Jensen, Professor and Vice Chair for Research, Department of Rehabilitation Medicine, University of Washington

Section 2. Search Terms used for databases

Medline (OVID); Ovid MEDLINE(R) ALL

1 exp Neuralgia/
2 neuralgia.mp.
3 (pain* adj3 neuropath*).mp.
4 (pain* adj3 polyneuropath*).mp.
5 (pain* adj1 radiculopath*).mp.
6 (spinal cord injur* adj3 pain).mp.
7 (nerve injur* adj3 pain).mp.
8 ((phantom or amputation) adj2 pain).mp.
9 (central post-stroke pain or central poststroke pain).mp.
1 or/1-9
11 exp Psychotherapy/
12 psychotherap*.mp.
13 exp Cognitive Behavioral Therapy/
14 cognitive behavio?r* therapy.mp.
15 exp Behavior Therapy/
16 behavio?r* therap*.mp.
17 cognitive therap*.mp.
18 exp Biofeedback, Psychology/
19 biofeedback.mp.
20 exp Meditation/
21 meditation.mp.
22 exp Mindfulness/
23 mindfulness.mp.
24 (psychological treatment* or psychological therap* or psychological intervention*).mp.
25 group therap*.mp.
26 self-regulation training.mp.
27 coping skill*.mp.
28 pain-related thought*.mp.
29 (behavio?r* adj2 rehabilitat*).mp.
30 ((psycho-education* or psychoeducation*) adj1 group*).mp.
31 exp mind-body therapies/ or relaxation therapy/
32 (relax* adj2 (technique* or therap*)).mp.
33 (mind adj2 body adj1 (therap* or technique*)).mp.
34 or/11-33
35 10 and 34

Embase (OVID); Embase Classic+Embase

1	exp neuralgia/ or neuropathic pain/
2	neuralgia.mp.
3	(pain* adj3 neuropath*).mp.
4	(pain* adj3 polyneuropath*).mp.
5	(pain* adj1 radiculopath*).mp.
6	(spinal cord injur* adj3 pain).mp.
7	(nerve injur* adj3 pain).mp.
8	phantom pain/
9	((phantom or amputation) adj2 pain).mp.
10	(central post-stroke pain or central poststroke pain).mp.
11	or/1-10
12	exp psychotherapy/
13	psychotherap*.mp.
14	exp cognitive behavioral therapy/
15	cognitive behavio?r* therapy.mp.
16	exp behavior therapy/
17	behavio?r* therap*.mp.
18	exp cognitive therapy/
19	cognitive therap*.mp.
20	exp biofeedback/
21	biofeedback.mp.
22	exp meditation/
23	meditation.mp.
24	exp mindfulness/
25	mindfulness.mp.
26	(psychological treatment* or psychological therap* or psychological intervention*).mp.
27	group therap*.mp.
28	self-regulation training.mp.
29	coping skill*.mp.
30	pain-related thought*.mp.
31	(behavio?r* adj2 rehabilitat*).mp.
32	((psycho-education* or psychoeducation*) adj1 group*).mp.
33	relaxation training/
34	(relax* adj2 (technique* or therap*)).mp.
35	(mind adj2 body adj1 (therap* or technique*)).mp.
36	or/12-35
37	11 and 36

PsycInfo (OVID); APA PsycInfo

1	exp Neuralgia/
2	neuralgia.mp.
3	neuropathic pain/
4	(pain* adj3 neuropath*).mp.
5	(pain* adj3 polyneuropath*).mp.
6	(pain* adj1 radiculopath*).mp.
7	(spinal cord injur* adj3 pain).mp.
8	(nerve injur* adj3 pain).mp.
9	((phantom or amputation) adj2 pain).mp.
10	(central post-stroke pain or central poststroke pain).mp.
11	or/1-10
12	exp Psychotherapy/
13	psychotherap*.mp.
14	exp cognitive behavior therapy/
15	cognitive behavio?r* therapy.mp.
16	exp behavior therapy/
17	behavio?r* therap*.mp.
18	cognitive therapy/
19	cognitive therap*.mp.
20	exp Biofeedback/
21	biofeedback.mp.
22	meditation/
23	meditation.mp.
24	mindfulness/
25	mindfulness.mp.
26	(psychological treatment* or psychological therap* or psychological intervention*).mp.
27	group therap*.mp.
28	self-regulation training.mp.
29	coping skill*.mp.
30	pain-related thought*.mp.
31	(behavio?r* adj2 rehabilitat*).mp.
32	((psycho-education* or psychoeducation*) adj1 group*).mp.
33	exp relaxation therapy/
34	(relax* adj2 (technique* or therap*)).mp.
35	Mind Body Therapy/
36	(mind adj2 body adj1 (therap* or technique*)).mp.
37	or/12-36
38	11 and 37

Cochrane Central Register of Controlled Trials (OVID)

1	exp Neuralgia/
2	neuralgia.mp.
3	(pain* adj3 neuropath*).mp.
4	(pain* adj3 polyneuropath*).mp.
5	(pain* adj1 radiculopath*).mp.
6	(spinal cord injur* adj3 pain).mp.
7	(nerve injur* adj3 pain).mp.
8	((phantom or amputation) adj2 pain).mp.
9	(central post-stroke pain or central poststroke pain).mp.
10	or/1-9
11	exp Psychotherapy/
12	psychotherap*.mp.
13	cognitive behavio?r* therapy.mp.
14	exp Behavior Therapy/
15	behavio?r* therap*.mp.
16	exp Cognitive Therapy/
17	cognitive therap*.mp.
18	exp Biofeedback, Psychology/
19	biofeedback.mp.
20	Meditation/
21	meditation.mp.
22	Mindfulness/
23	mindfulness.mp.
24	(psychological treatment* or psychological therap* or psychological intervention*).mp.
25	group therap*.mp.
26	self-regulation training.mp.
27	coping skill*.mp.
28	pain-related thought*.mp.
29	(behavio?r* adj2 rehabilitat*).mp.
30	((psycho-education* or psychoeducation*) adj1 group*).mp.
31	exp mind-body therapies/ or relaxation therapy/
32	(relax* adj2 (technique* or therap*)).mp.
33	(mind adj2 body adj1 (therap* or technique*)).mp.
34	or/11-33
35	10 and 34

CINAHL Complete

S1	(MH "Neuralgia+")
S2	neuralgia
S3	TX(pain* N3 neuropath*)
S4	TX(pain* N3 polyneuropath*)
S5	TX(pain* N1 radiculopath*)
S6	TX(spinal cord injur* N3 pain)
S7	TX(nerve injur* N3 pain)
S8	(MH "Phantom Pain")
S9	TX((phantom OR amputation) N2 pain)
S10	"Central post-stroke pain" OR "central poststroke pain"
S11	S1 OR S2 OR S3 OR S4 OR S5 OR S6 OR S7 OR S8 OR S9 OR S10
S12	(MH "Psychotherapy+")
S13	psychotherap*
S14	"cognitive behavio#r* therapy"
S15	(MH "Behavior Therapy+")
S16	"behavio#r* therap*"
S17	(MH "Cognitive Therapy+")
S18	"cognitive therap*"
S19	(MH "Biofeedback")
S20	biofeedback
S21	(MH "Meditation")
S22	meditation
S23	(MH "Mindfulness")
S24	mindfulness
S25	"psychological treatment*" OR "psychological therap*" OR "psychological intervention*"
S26	"group therap*"
S27	"self-regulation training"
S28	"coping skill*"
S29	"pain-related thought*"
S30	TX(behavio#r* N2 rehabilitat*)
S31	TX((psycho-education* OR psychoeducation*) N1 group*)
S32	(MH "Mind Body Techniques+") OR (MH "Relaxation Techniques+")
S33	TX(relax* N2 (technique* OR therap*))
S34	TX(mind N2 body N1 (therap* or technique*))
S35	S12 OR S13 OR S14 OR S15 OR S16 OR S17 OR S18 OR S19 OR S20 OR S21 OR S22 OR S23 OR S24 OR S25 OR S26 OR S27 OR S28 OR S29 OR S30 OR S31 OR S32 OR S33 OR S34
S36	S11 AND S35

Scopus

(TITLE-ABS-KEY (neuralgia OR (pain* W/3 neuropath*) OR (pain* W/3 polyneuropath*) OR (pain* W/1 radiculopath*) OR (spinal AND cord AND injur* W/3 pain) OR (nerve AND injur* W/3 pain) OR ((phantom OR amputation) W/2 pain) OR "central post-stroke pain" OR "central poststroke pain")) AND ((TITLE-ABS-KEY (psychotherap* OR "cognitive behavio#r* therapy" OR "behavio#r* therap*" OR "cognitive therap*" OR biofeedback OR meditation OR mindfulness OR "psychological treatment*" OR "psychological therap*" OR "psychological intervention*" OR "group therap*") OR TITLE-ABS-KEY ("self-regulation training" OR "coping skill*" OR "pain-related thought*" OR (behavio#r* W/2 rehabilitat*) OR ((psycho-education* OR psychoeducation*) W/1 group*) OR (relax* W/2 (technique* OR therap*)) OR (mind W/2 body W/1 (therap* OR technique*))))))

Web of Science Core Collections

Neuralgia OR (pain* NEAR/3 neuropath*) OR (pain* NEAR/3 polyneuropath*) OR (pain* NEAR/1 radiculopath*) OR (spinal cord injur* NEAR/3 pain) OR (nerve injur* NEAR/3 pain) OR ((phantom OR amputation) NEAR/2 pain) OR “central post-stroke pain” OR “central poststroke pain” (Topic) and psychotherap* OR “cognitive behavio$r* therapy” OR “behavio$r* therap*” OR “cognitive therap*” OR biofeedback OR meditation OR mindfulness OR "psychological treatment*" OR "psychological therap*" OR "psychological intervention*" OR “group therap*” OR “self-regulation training” OR “coping skill*” OR “pain-related thought*” OR (behavio$r* NEAR/2 rehabilitat*) OR ((psycho-education* OR psychoeducation*) NEAR/1 group*) OR (relax* NEAR/2 (technique* OR therap*)) OR (mind NEAR/2 body NEAR/1 (therap* OR technique*)) (Topic)
